# EPA guidance on the quality of mental health services: A systematic meta-review and update of recommendations focusing on care coordination

**DOI:** 10.1192/j.eurpsy.2020.75

**Published:** 2020-07-24

**Authors:** W. Gaebel, A. Kerst, B. Janssen, T. Becker, M. Musalek, W. Rössler, M. Ruggeri, G. Thornicroft, J. Zielasek, J. Stricker

**Affiliations:** 1 Department of Psychiatry, Medical Faculty, LVR-Klinikum Düsseldorf, Heinrich-Heine-University, Düsseldorf, Germany; 2 WHO Collaborating Centre on Quality Assurance and Empowerment in Mental Health, Düsseldorf, Germany; 3 LVR-Klinik Langenfeld, Langenfeld, Germany; 4 Department of Psychiatry II, University of Ulm, Bezirkskrankenhaus Günzburg, Germany; 5 Anton Proksch Institute, Vienna, Austria; 6 Department of Psychiatry and Psychotherapy, Charité, Universitätsmedizin Berlin, Berlin, Germany; 7 Psychiatric University Hospital, University of Zurich, Zurich, Switzerland; 8 Laboratory of Neuroscience (LIM 27), Institute of Psychiatry, University of Sao Paulo; Sao Paulo, Brazil; 9 Section of Psychiatry, Department of Public Health and Community Medicine, University of Verona, Verona, Italy; 10 Centre for Global Mental Health, Institute of Psychiatry, Psychology &Neuroscience, King’s College, London, United Kingdom; 11 LVR-Institute for Healthcare Research, Cologne, Germany

**Keywords:** Care coordination, integrated care, mental health, quality

## Abstract

**Background.:**

The quality of mental health services is crucial for the effectiveness and efficiency of mental healthcare systems, symptom reduction, and quality of life improvements in persons with mental illness. In recent years, particularly care coordination (i.e., the integration of care across different providers and treatment settings) has received increased attention and has been put into practice. Thus, we focused on care coordination in this update of a previous European Psychiatric Association (EPA) guidance on the quality of mental health services.

**Methods.:**

We conducted a systematic meta-review of systematic reviews, meta-analyses, and evidence-based clinical guidelines focusing on care coordination for persons with mental illness in three literature databases.

**Results.:**

We identified 23 relevant documents covering the following topics: case management, integrated care, home treatment, crisis intervention services, transition from inpatient to outpatient care and vice versa, integrating general and mental healthcare, technology in care coordination and self-management, quality indicators, and economic evaluation. Based on the available evidence, we developed 15 recommendations for care coordination in European mental healthcare.

**Conclusions.:**

Although evidence is limited, some concepts of care coordination seem to improve the effectiveness and efficiency of mental health services and outcomes on patient level. Further evidence is needed to better understand the advantages and disadvantages of different care coordination models.

## Introduction

The quality of mental health services plays a central role for the effectiveness and efficiency of mental healthcare systems, symptom reduction, and quality of life improvements on patient level [1]. Thus, a 2012 European Psychiatric Association (EPA) guidance provided evidence-based recommendations for optimal mental health services in Europe [2]. Quality is a multidimensional construct that can be defined according to various components and dimensions (e.g., categories or levels of observation; see [3]). Quality monitoring, assurance, and improvement assessment in mental healthcare are quickly growing fields of research. Hence, an update of the EPA guidance reflecting the current state of the empirical literature is needed. In this updated EPA guidance, we focus specifically on care coordination in mental healthcare. The aim of care coordination is to provide efficient and patient-centered care across different mental health services and other health services at the interface with the aim to improve health outcomes [4]. A number of models and concepts can be subsumed under the umbrella term “care coordination.” In the scope of this manuscript, we will first briefly describe a selection of concepts in the context of care coordination. The strict differentiation between the various concepts is a rather theoretical approach. In practice, they overlap, and exact boundaries between them cannot be drawn [5]. Next, we present results from a systematic literature review of systematic reviews, meta-analyses, and evidence-based clinical guidelines on care coordination. Based on these results, we then developed recommendations for care coordination in mental healthcare that are graded based on the available evidence.

### Care Coordination: Models and Concepts

Care coordination involves the integration of care across the patient’s different needs and conditions during the illness course, across different providers and treatment settings (e.g., inpatient and outpatient care), according to the patient’s capacity and preferences [6]. Integrated care is often used synonymously for care coordination and can be described as “the management and delivery of health services so that clients receive a continuum of preventive and curative services, according to their needs over time and across different levels of the health system” [7]. Both concepts describe instruments to improve services in relation to access, quality, user satisfaction, and efficiency [7].

The reduction of institutionalized care and length of hospital stays has a high priority in models of care coordination [8]. Community-based care is a form of care coordination that adopts a decentralized care pattern to decrease the duration of inpatient care. This concept promotes mental health for local populations including a wide network of support, services, and resources of adequate capacity [9]. Team-based approaches (e.g., home treatment teams or crisis resolution teams) are widely implemented in community-based care concepts to respond to acute mental health difficulties by providing intensive home-based treatment and support. Care coordination across different settings involves the collaboration of various care providers and patients, for example, between mental health specialists and other care providers. Consultation liaison psychiatry, for example, has a strong history in hospital-based care but is becoming increasingly important in primary care settings [10].

To address the individual coordination of care for certain patient groups, the case management model has emerged [11]. This model aims to integrate care across a variety of health services for individuals with complex needs [12]. Case management implicates a collaborative process that aims to ensure a continuum of care through effective resource coordination [11]. Although there are some variations of this model, all current models of case management include the assignment of a case manager in order to achieve effective care coordination for the patient. Another model that has partly emerged from case management is Intensive Case Management (ICM). This approach contains community-based healthcare services for individuals with severe mental illness who do not require immediate admission [13]. The caseload in this model is usually smaller, and the intensity of support is higher than in regular case management models [13]. For more elaborate definitions of types of mental health services, please also refer to the preceding guidance paper [2].

Integrated health services depend on the interaction of different care providers. This model requires the accessibility of health services at the local level. However, availability of specialty services might be limited, for example, in rural areas. Therefore, the interest in using digital technology in the provision of health services has considerably increased in recent years. Information and communication technologies can support the remote management of healthcare, for example, by providing self-management tools or enabling electronic communication between service providers and users across distances [14]. Proponents consider the flexibility of digital solutions in mental healthcare as especially suitable for integrated- and community-based care settings and in chronic patients [15]. When implementing coordinated care models, their cost-effectiveness and the assurance of adequate quality of mental health services by measuring quality indicators are also important. Therefore, this meta-review also includes documents that focus on such quality indicators and the cost-effectiveness of care coordination.

### The Present Study

The aim of this guidance was to update recommendations regarding care coordination across different mental healthcare services. We systematically reviewed the available evidence from systematic reviews, meta-analyses, and evidence-based clinical guidelines on care coordination in mental healthcare that have been published since the last EPA guidance on the quality of mental health services in Europe [2]. Additionally, we developed recommendations based on the available empirical evidence.

## Methods

### Guidance development

This guidance was developed in accordance with the EPA guidance framework (for details see [16]). The quality of mental health services and care coordination are extensive and multifaceted topics. Thus, we conducted a systematic meta-review (i.e., a systematic overview of meta-analyses, systematic reviews, and evidence-based clinical guidelines) rather than a review of all available primary studies. With this approach, we focus on research that provides high levels of evidence.

### Search strategy and inclusion criteria

We conducted a standardized literature search in the databases Medline (PubMed), Scopus, and the Cochrane Database of Systematic Reviews applying the search string (“care coordination” OR “case management” OR “integrated care” OR “coordinated care” OR “community based care” OR “home treatment” OR “managed care”) AND (psychiatr* OR “mental”) in titles, abstracts, and keywords in March 2020. We limited study inclusion to systematic review articles, meta-analyses, and evidence-based clinical guidelines. To assure that only studies that were published after the first EPA guidance on the quality of mental health services are included, we set 2011 as the date limit for the inclusion of documents. We applied the following inclusion and exclusion criteria: (a) Studies had to aggregate findings from quantitative investigations of care coordination for persons with mental illness. (b) Studies had to be published in English. (c) Studies focusing on children/adolescents, somatic comorbidity, or a specific mental disorder or symptom (rather than people with mental illness more generally) were excluded.

### Study selection and quality assessment

**Figure 1.**
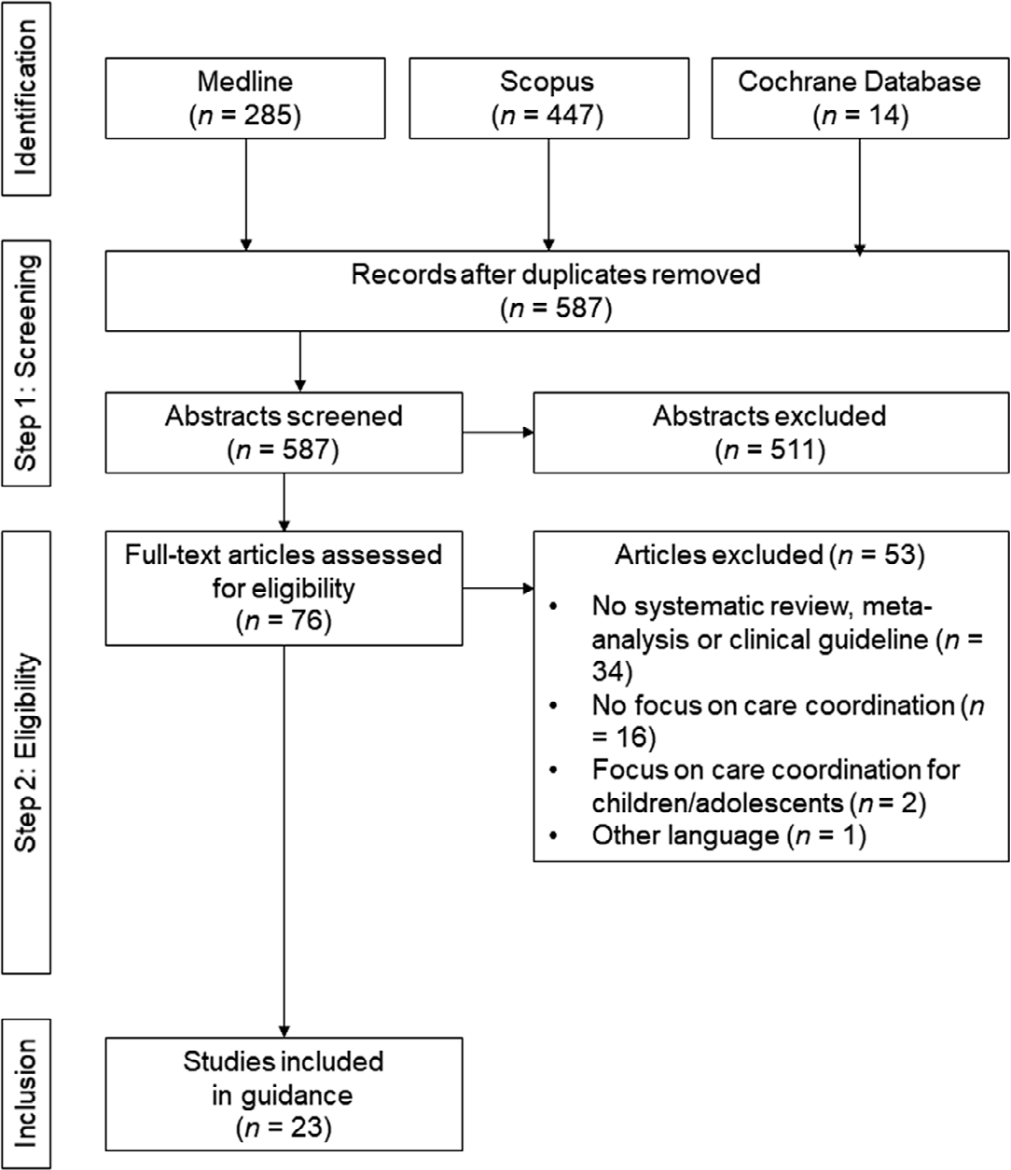
Flow diagram of the study search and inclusion process.

Figure 1 displays the flow diagram depicting the study search and inclusion process. We determined study eligibility in two steps. In the first step, two independent raters (A.K. and J.S.) screened the titles and abstracts of all studies identified in the systematic literature search (*n* = 587 after removing duplicates) and decided to either exclude or retain the respective article for inspection of the full text (e.g., articles that clearly indicated a focus on children or adolescents in the article title were excluded in this step). In the second step, both raters independently determined study eligibility for the remaining studies (*n* = 76) based on the full texts. Agreement ([number of consistently coded studies/total number of coded studies] × 100) was 91.1% in Step 1 and 94.7% in Step 2. All disagreements were resolved by consulting the original study manuscripts. Finally, each of the two raters independently excerpted the thematic focus, methods, and main findings for 50% of the included studies. For the respectively coded studies, each rater additionally applied the AMSTAR 2 checklist [17]. The AMSTAR 2 checklist is a tool for the critical appraisal of systematic reviews of healthcare interventions. Raters evaluate the quality of a systematic review or meta-analysis on 16 items, of which only three apply to meta-analyses. In accordance with prior research, we computed an overall numerical value for each included study by scoring all items whose criteria were fully fulfilled by a study as “1” and all other items as “0.” The maximum possible AMSTAR 2 score for meta-analyses (i.e., 16) is higher than for systematic reviews (i.e., 13). To allow comparison of the AMSTAR 2 scores across studies with different methodologies, we transformed the AMSTAR 2 raw scores to percentages of maximum possible scores (POMP scores [18]). Next, we rated the grade of evidence for each systematic review, meta-analysis, or evidence-based clinical guideline on a 4-point scale based on the AMSTAR 2 ratings. Table 1 displays the grading system.

**Table 1.** Grade of evidence for systematic reviews and meta-analyses*.*

Grade	Description
1	High-quality meta-analyses, systematic reviews of RCTs, or evidence-based clinical guideline with a very low risk of bias (AMSTAR 2 ratings 100–80%).
2	Well-conducted meta-analyses, systematic reviews, or evidence-based clinical guidelines with a low risk of bias (AMSTAR 2 ratings 80–60%).
3	Meta-analyses, systematic reviews, or clinical guidelines with an increased risk of bias (AMSTAR 2 ratings 60–40%).
4	Meta-analyses, systematic reviews, or clinical guidelines with a considerable risk of bias (AMSTAR 2 ratings 40–0%).

Abbreviation: RCT, randomized controlled trial.

### Development and grading of recommendations

The topic of this guidance was approved by the EPA guidance committee. We developed the recommendations based on the available evidence in a consensus process involving all authors of this manuscript who represent a substantial proportion of European experts in care coordination. The recommendations were graded on a 4-point scale according to the evidence available to support the respective recommendation (see Table 2).

**Table 2.** Grading of guidance recommendations (modified from [19]).

Recommendation grade	Description
A	At least one meta-analysis, systematic review, or evidence-based clinical guideline with clear findings, rated as 1 and directly applicable to the target population.
B	At least one meta-analysis, systematic review, or evidence-based clinical guideline rated as 2 aggregating a body of evidence from primary studies that are directly applicable to the target population and demonstrate overall consistency of results.
C	At least one meta-analysis, systematic review, or evidence-based clinical guideline rated as 3 or 4 aggregating a body of evidence that demonstrates overall consistency of results or evidence from meta-analysis, systematic review, or evidence-based clinical guideline rated as 1 or 2 but reporting limited evidence or less consistent findings regarding the respective recommendation (e.g., a significant overall trend but substantial heterogeneity).
D	Good practice recommendations based on the clinical experience of the guidance development group (expert consensus).

## Results

### Study characteristics

Overall, we identified 23 relevant documents (16 systematic reviews, 6 meta-analyses, and 1 evidence-based clinical guideline). The median publication year was 2015. Table 3 displays the methods, main findings, AMSTAR 2, and evidence-level ratings for all included studies. AMSTAR 2 ratings were relatively low which is, in some cases, due to the thematic focus of the included studies (e.g., randomized controlled trials [RCTs] with control conditions are more difficult to realize for cost-effectiveness analyses than for other interventions in mental healthcare).

**Table 3.** Focus, methods, main results, and quality ratings of the included studies (*n* = 23).

Study		Focus	Methods	Main results	AMSTAR 2	Evidence level rating
	I. Case management, integrated mental health services, and home treatment					
Dieterich et al. [12]		Intensive Case Management (ICM) for severe mental illness	Cochrane systematic review. Meta-analysis including 40 randomized controlled trials (RCTs).	In comparison to standard care, persons with ICM were more likely to stay with the service, had improved general functioning, had a higher chance to find employment and to be not homeless, and had shorter stays in hospital (especially for those with long previous inpatient periods). In comparison to nonintensive case management, the only clear difference was ICM reduced the number of persons leaving the intervention.	94%	1
Gühne et al. [20]		Psychosocial therapies in severe mental illness	S3-Guideline developed in a systematic evidence-based process including a literature review and expert consensus.	Good evidence for the efficacy of the majority of psychosocial interventions. Best available evidence for multidisciplinary team–based psychiatric community care, family psychoeducation, social skills training, and supported employment.	77%	2
Karapareddy [21]		Integrated care for combined mental health and substance use disorders	Literature review and meta-analysis. Twelve studies were included for quantitative data synthesis. Three of those studies were used for an analysis of cost-effectiveness.	Models of care that integrate treatment for combined substance use and mental illness are more effective than conventional, non-integrated models. Integrated models are superior to standard care models through reductions in substance use and improvement of mental health. Integrated models are more cost-effective than standard care. Overall, the evidence is limited.	19%	4
Klug et al. [22]		Multidisciplinary psychiatric home treatment for elderly patients with mental illness	Systematic literature review in several databases and hand searches. Three studies were included in the review.	Psychogeriatric home treatment is associated with significant improvements of psychiatric symptoms and psychosocial problems, fewer admissions to hospital and nursing homes, and lower costs of care. Overall, there is limited evidence.	77%	2
Thomas and Rickwood [23]		Clinical and cost-effectiveness of acute and subacute residential mental health services	Systematic review based on a search in four databases. Inclusion of 26 studies (9 RCTs)	Overall, clinical outcomes for persons in acute residential mental health services were equal to those of persons in inpatient treatment, with similar readmission rates and higher cost-effectiveness and higher user satisfaction in acute residential treatment compared to inpatient treatment. The number of studies in subacute residential mental health services was too low to draw conclusions.	15%	4
Wright-Berryman et al. [24]		Consumer-provided services in assertive community treatment and ICM teams for adults with severe mental illness	Systematic review based on a meta-search of eight databases. Inclusion of 16 studies that report consumer-level outcomes (8 RCTs)	The inclusion of persons that have experienced mental illness in teams for assertive community treatment or ICM improves treatment engagement but not clinical outcomes. Overall, the evidence is limited.	38%	3
	II. Crisis intervention services					
Carpenter et al. [25]		Effectiveness of crisis resolution teams (CRTs) in practice	Systematic literature review of RCTs and non-randomized studies. 37 studies were included.	CRTs appear effective in reducing admissions. However, data are mixed, and other factors may also have an influence. Evidence of CRT on compulsory admissions is inconclusive. There are few clinical differences between “gate-kept” patients admitted and those that were not. CRTs are saving costs compared to inpatient care. Patients are satisfied with CRT care. Overall, high-quality evidence for CRT is scarce.	31%	3
Murphy et al. [26]		Crisis intervention for people with severe mental illnesses	Cochrane review. Meta-analysis including eight RCTs.	Care based on crisis-intervention principles, with or without an ongoing homecare package, appears to be a viable and acceptable way of treating people with serious mental illnesses. Evidence is limited and of low to moderate quality.	100.00%	1
Paton et al. [27]		Mental health crisis services (with a focus on the United Kingdom)	Rapid systematic review of guidelines and high-quality primary studies based on a search in different databases and hand search. 16 systematic reviews and 15 primary studies were included.	Telephone support and triage appear to result in quick access before the crisis point. Liaison psychiatry may reduce readmission rates and waiting times and improve service user satisfaction. Crisis resolution and home treatment teams are clinically effective and cost-effective. Crisis houses and acute day hospitals were not more clinically effective than inpatient treatment. Overall, the evidence is limited. Particularly, there is a lack of RCTs.	77%	2
Toot et al. [28]		Effectiveness of crisis resolution/home treatment teams for older people with mental illness	Systematic review based on a search in three databases. Inclusion of 10 documents, none of which was a randomized controlled trial.	Overall, the evidence is limited. Based on the very little robust evidence, crisis resolution/home treatment teams for older people with mental health problems reduce the number of admissions to hospital.	31%	3
Wheeler et al. [29]		CRT models for adults with mental illness who would otherwise be admitted to inpatient care	Systematic review based on a search in five databases and an additional web-based search. Inclusion of 69 studies with varying quality.	No confident conclusions can be drawn about the critical components of CRTs due to limited evidence. There was some empirical support for the inclusion of a psychiatrist in the CRT and provision of a 24-h service (rather than shorter operating hours).	62%	2
	III. Transition from inpatient to outpatient care and vice versa, return to work					
Clibbens et al. [8]		Early discharge in acute mental healthcare	Rapid literature review in different databases and hand searches. 14 studies were included (7 reported quantitative data, 3 reported qualitative data, and 4 reported mixed-methods data).	Early discharge was not limited to crisis resolution and home treatment (CRHT). Studies showed that discharge planning is required. Early discharge was not associated with unplanned readmissions and had a small effect on length of stay. Most studies reported service outcomes. Health outcomes were underreported. Professionals and service users were positive about early discharge. Carers preferred hospital or day hospital care. Overall, the evidence is limited and leaves an unclear picture of early discharge.	31%	3
Hegedüs et al. [30]		Transitional interventions in improving patient outcomes and service use after discharge from psychiatric inpatient care	Systematic review based on database searches. Random effects meta-analysis of 9 RCTs (overall 16 studies: 10 RCTs, 3 quasi-experimental, 3 cohort studies).	Interventions included components from case management, psychoeducation, cognitive behavioral therapy, and peer support. All studies with significant improvements in at least one outcome provided elements of case management Transitional interventions with bridging components were no more effective in reducing readmission than treatment as usual. Overall, the evidence is limited.	94%	1
MacEachen et al. [31]		Return to work (RTW) coordinators for people affected by common mental illness	Scoping review of qualitative or quantitative, mixed methods, or scoping and systematic review articles. 5 quantitative studies were included.	Findings suggest that interventions for mental ill-health that employs RTW coordinators may be more time consuming than conventional approaches and may not increase RTW rate or worker’s self-efficacy for RTW. The evidence base is limited.	38%	3
Tricco et al. [32]		Impact of quality improvement strategies for coordination of care on hospital admission rates in different patient groups (including patients with mental illness)	Systematic review and meta-analysis based on a literature search in three databases and further hand search. Inclusion of 50 studies (36 RCTs, 14 companion reports)	No impact of quality improvement strategies (e.g., case management, team changes, promotion of self-management, decision support, clinical information systems) on hospital admission in patients with mental illness. This may be due to characteristics of the control groups (i.e., frequent care coordination strategies as part of the control condition).	63%	2
	IV. Integrating general and mental healthcare					
Bradford et al. [33]		Interventions that integrate medical and mental healthcare to improve general medical outcomes in individuals with serious mental illness	Systematic review of RCTs and quasi-experimental studies based on a search in different databases and hand searches (overall 4 RCTs were included).	Integrated care models have positive effects on processes of preventive and chronic care. Results on physical functioning in individuals with serious mental illness are mixed. Overall, there is a small number of trials available for integrated treatment models.	54%	3
Gillies et al. [10]		Consultation liaison in primary care	Cochrane review. Meta-analysis including 11 RCTs.	Consultation liaison improves mental health for up to 3 months and satisfaction and adherence for up to 12 months in persons with mental illness, particularly in individuals with depression. Care providers were more likely to provide adequate treatment and prescribe pharmacological therapy. Consultation liaison may not be as effective as collaborative care. The overall quality of trials was low.	94%	1
Oldham et al. [34]		Proactive psychiatric consultation in general hospitals	Systematic review based on a search of four databases. 12 studies (2 RCTs) were included.	Proactive consultation liaison psychiatry (i.e., psychiatrists working within medical or surgical settings or multidisciplinary team-based models) with clinically informed screening and integrated care delivery reduces length of stay in general hospital settings. High-quality evidence was limited.	46%	3
	V. Technology in care coordination and self-management					
Falconer et al. [35]		Use of technology for care coordination	Systematic literature review in different databases. 21 articles were included.	Electronic health records were most commonly used for care coordination. Care coordination provided easier patient access to healthcare and improved communication between the caregiver and patient, especially when geographic distance is a challenge. Barriers included insufficient funding for health information technology, deficient reimbursement plans, limited access to technologies, cultural barriers, and underperforming electronic health record templates.	38%	3
Kelly et al. [36]		Self-management healthcare models for individuals with serious mental illnesses	Systematic literature review in several databases and hand searches. 14 studies were included (10 RCTs and 4 within-person pre–post designs).	Individuals with serious mental health issues can collaborate with health professionals or be trained to self-manage their general health and healthcare. The evidence supports the use of mental health peers or professional staff to implement healthcare interventions. Overall, there is limited evidence and there is large heterogeneity in study results.	46%	3
	VI. Quality indicators and economic evaluation					
Goldman et al. [37]		Quality indicators for integrated care	Systematic database search for quality indicators and additional literature review.	Quality measures predominantly concentrate on care during or following hospitalizations, which represents a minority of behavioral healthcare and does not characterize the outpatient settings that are the focus of many models of integrated care.	46%	3
Knapp et al. [38]		Economic consequences of deinstitutionalization of mental health services	Systematic literature review in several databases and expert opinion.	Community-based models of care are not inherently costlier than institutions, when individuals’ needs and the quality of care are taken into account. Community-based care could be more expensive than long-stay hospital care but may still be seen as more cost-effective because, when set up and managed well, they deliver superior outcomes.	15%	4
Sunderji et al. [39]		Quality indicators for integrated mental healthcare	Systematic review based on a search of five databases and gray literature sources. 172 literature sources were included.	148 unique quality measures were distilled. There are quality measures based on evidence-based care processes, individual clinical outcomes, efficiency (cost-effectiveness), and client satisfaction. Measures of safety of care, equitability, accessibility, and timeliness of care are largely missing.	54%	3

We computed a score for AMSTAR 2 by scoring all items whose criteria were fully fulfilled by a study as “1” and all other items as “0” and by transforming the raw scores into percentage of maximum possible scores (POMP scores [18]).

Study samples included individuals with severe mental illness (e.g., schizophrenia and psychosis, severe mood problems), substance use disorder, anxiety disorder, geriatric patients with mental illness (mood disorders, schizophrenia spectrum disorders, cognitive impairment), individuals with psychosis, bipolar disorder, or self-harm-experiencing mental health crisis. Outcome measures were related to health outcomes, for example, symptom reduction, global functioning, quality of life, or service outcomes, for example, service satisfaction, number of admissions, or costs of care.

Despite some overlap, we have categorized the included studies according to six thematic topics in an inductive process. The studies compare either several components of coordinated care models or focus on one specific coordinated care component: (a) Case management, integrated mental health services, and home treatment, (b) Crisis intervention services, (c) Transition from inpatient to outpatient care and vice versa, return to work, (d) Integrating general and mental healthcare, (e) Technology and self-management in care coordination, and (f) Quality indicators and economic evaluation.

## Recommendations

Based on the systematic meta-review of systematic reviews, meta-analyses, and evidence-based clinical guidelines, we developed 15 recommendations for care coordination in mental healthcare services in a consensus process. For comparison with previous recommendations, please refer to Table 1 of the initial EPA Guidance on the Quality of Mental Health Services [2].

## General recommendations

*Recommendation 1. Research programs on mental healthcare services are needed that systematically assess the impact of different models of care coordination on patient-level and healthcare system–level outcomes (recommendation grade D).* The majority of systematic reviews and meta-analyses included in this systematic meta-review concluded that the currently available evidence is insufficient to derive definite conclusions. Thus, additional research is needed and should be adequately funded by the relevant national and international funding bodies.

### Case management, integrated care, and home treatment

*Recommendation 2. Implement Intensive Case Management for people with severe mental illness who are high users of inpatient care and difficult to engage or recurrently disengage (recommendation grade B).* ICM may lead to shorter inpatient treatment, lower drop-out rates, and improved social functioning in severely mentally ill persons compared to standard care (i.e., simple outpatient appointments) [13]. Compared to nonintensive case management, intensive case management is associated with lower drop-out rates [13] (compare [2], recommendation 15)” (p. 11) in comparison to standard care for severely mentally ill persons [2]. The recommendation is confirmed based on an update of the review and meta-analysis of Dieterich et al. ([40]; compare [2], recommendation 15).

*Recommendation 3. Implement multidisciplinary team–based psychiatric community care (recommendation grade B).* There is good evidence for the effectiveness of psychiatric community care for improving various outcomes [20]. Teams for community or home-based treatment should be multidisciplinary, comprising psychiatric, psychological, and other members of the mental health work force (e.g., nursing staff). Based on updated research, the recommendations to develop a system of community mental health teams (compare [2], recommendation 14) and to assemble multiprofessional teams in service provision are confirmed (compare [2], recommendation 4).

*Recommendation 4.* *Include persons who have experienced mental illness in teams for community-based treatment for persons with severe mental illness (recommendation grade D).* Although the inclusion of persons who have experienced mental illness in teams for community-based treatment does not improve clinical outcomes, it may increase treatment engagement in persons with severe mental illness [24].

*Recommendation 5. Offer integrated care for persons with combined mental health and substance use disorders (recommendation grade C).* Integrated models of care are more cost-effective and more effective in reducing substance abuse and improving mental health in persons with combined mental health and substance use disorders than conventional, nonintegrated models [21]. This recommendation also builds on a previous recommendation to implement integrated care (compare [2], recommendation 16).

*Recommendation 6. Provide multidisciplinary psychogeriatric home treatment for elderly persons with mental illness (recommendation grade C).* Psychogeriatric home treatment is associated with significant improvements in psychiatric symptoms and psychosocial problems, fewer admissions to hospital and nursing homes, and lower costs of care compared to care as usual [22]. Due to the increased somatic comorbidity in elderly persons with mental illness, psychogeriatric home treatment teams should involve medical experts.

### Crisis intervention services

*Recommendation 7. Offer acute and subacute residential mental health services as an alternative to inpatient treatment for patients whose mental condition does not necessarily require inpatient treatment (recommendation grade C).* Clinical outcomes (including readmission rates) do not seem to differ significantly between persons with mental illness in residential treatment and hospital inpatient treatment, but residential treatment is associated with higher cost-effectiveness. Additionally, user satisfaction is higher in acute residential treatment compared to inpatient treatment [23].

*Recommendation 8. Implement crisis intervention teams for people with mental illness in home and community treatment (recommendation grade B).* Crisis intervention teams (or crisis resolution teams) are a viable and cost-effective alternative to inpatient treatment [25,26,27] that may reduce admission rates [25,26] and waiting times and increase service user satisfaction [27]. Crisis intervention teams were also considered effective components of home treatment previously (compare [2], recommendation 27).

*Recommendation 9. Offer crisis interventions teams as a 24 h-service and include a psychiatrist in crisis intervention teams in mental healthcare (recommendation grade C).*

Although evidence is rater limited, operating crisis resolution teams as 24-h services (rather than shorter operating hours) and including a psychiatrist in crisis resolution teams seem to be associated with increased effectiveness [29].

### Transition from inpatient to outpatient care and vice versa

*Recommendation 10. Provide elements of case management to persons with mental illness after discharge from inpatient treatment (recommendation grade C).* Overall, a systematic review found no positive effect of transitional interventions on readmission rates compared to treatment as usual. Yet, there was some limited evidence that elements of case management (e.g., transition managers and timely communication between inpatient staff and outpatient care) may have positive effects on health-related and social outcomes (e.g., symptom severity, quality of life). Additionally, service users prefer transitional interventions [30].

### Integrating general and mental healthcare

*Recommendation 11. Implement consultation liaison psychiatry in primary healthcare (recommendation grade B).* Psychiatric consultation liaison services in primary healthcare improve mental health for up to 3 months and satisfaction and adherence for up to 12 months in persons with mental illness. This effect is particularly strong for persons with depression [10]. Additionally, proactive consultation liaison psychiatry (i.e., psychiatrists working in medical or surgical hospital settings or multidisciplinary team–based models) with clinically informed screening and integrated care delivery reduce length of stay in general hospital settings [34]. Consultation liaison psychiatry should be implemented in both inpatient and outpatient medical healthcare.

*Recommendation 12. Offer integrated care models for persons with mental illness to improve general medical outcomes (recommendation grade C).* Incorporating medical preventive and chronic disease care in mental healthcare improves rates of immunization and screening for medical disorders accompanied by positive effects on physical health [33].

### Technology in care coordination and self-management

*Recommendation 13. Use digital technology such as electronic health records to enhance care coordination (recommendation grade C/D).* Care coordination with electronic health records provides easier patient access to healthcare and improves communication between the caregiver and patient [35]. Sufficient funding, reimbursement, and access to technologies must be secured.

*Recommendation 14. Provide persons with severe mental illness with trainings to efficiently self-manage their general health and healthcare (recommendation grade C).* There is some evidence that mental health professionals or peers (i.e., persons with a history of mental illness) may efficiently provide self-management training (e.g., comprising action planning) that improves general health outcomes in persons with severe mental illness [36]. These interventions should be offered as a supplement to existing models of care.

### Quality indicators and economic evaluation

*Recommendation 15. Systematically develop and implement quality indicators for integrated care models across mental health services (recommendation grade D).* There are various quality indicators for integrated care models in mental healthcare, particularly for evidence-based care processes, individual clinical outcomes, efficiency (cost-effectiveness), and client satisfaction [38]. However, quality indicators assessing safety of care, equitability, accessibility, and timeliness of care [39], as well as quality indicators that focus on outpatient settings as provided by models of integrated care [37] are largely lacking. We consider suggestions for quality indicators from the preceding guidance paper [2] as valid.

## Discussion and Conclusions

In this meta-review, we summarized systematic reviews, meta-analyses, and evidence-based clinical guidelines on models of care coordination for persons with mental illness. Although we identified a substantial number of relevant documents, evidence was weak in that most included systematic reviews and meta-analyses concluded that findings from the available primary studies were insufficient to draw definitive conclusions. A lack of robust evidence had also been identified in the previous guidance, which explains why the recommendations were based mainly on expert opinion [2]. In this update of the initial EPA guidance, stronger evidence was available for some but not for all aspects of integrated care. Thus, future research is needed that disentangles the unique and interactive effects of the various components of integrated care. To further improve the evidence base for integrated care, we urgently recommend empirical monitoring and evaluation of the various care coordination projects in mental healthcare that will be implemented in the coming years in Europe and elsewhere.
